# Estimating Waterborne Infectious Disease Burden by Exposure Route, United States, 2014

**DOI:** 10.3201/eid2907.230231

**Published:** 2023-07

**Authors:** Megan E. Gerdes, Shanna Miko, Jasen M. Kunz, Elizabeth J. Hannapel, Michele C. Hlavsa, Michael J. Hughes, Matthew J. Stuckey, Louise K. Francois Watkins, Jennifer R. Cope, Jonathan S. Yoder, Vincent R. Hill, Sarah A. Collier

**Affiliations:** Chenega Corporation, Atlanta, Georgia, USA (M.E. Gerdes);; Centers for Disease Control and Prevention, Atlanta (M.E. Gerdes, S. Miko, J.M. Kunz, E.J. Hannapel, M.C. Hlavsa, M.J. Hughes, M.J. Stuckey, L.K. Francois Watkins, J.R. Cope, J.S. Yoder, V.R. Hill, S.A. Collier)

**Keywords:** waterborne infections, Cryptosporidium, Giardia, Legionnaires’ disease, mycobacteria, Pseudomonas, disease burden, opportunistic premise plumbing pathogens, waterborne pathogens, bacteria, enteric infections, parasites, tuberculosis and other mycobacteria, viruses, zoonoses, United States

## Abstract

More than 7.15 million cases of domestically acquired infectious waterborne illnesses occurred in the United States in 2014, causing 120,000 hospitalizations and 6,600 deaths. We estimated disease incidence for 17 pathogens according to recreational, drinking, and nonrecreational nondrinking (NRND) water exposure routes by using previously published estimates. In 2014, a total of 5.61 million (95% credible interval [CrI] 2.97–9.00 million) illnesses were linked to recreational water, 1.13 million (95% CrI 255,000–3.54 million) to drinking water, and 407,000 (95% CrI 72,800–1.29 million) to NRND water. Recreational water exposure was responsible for 36%, drinking water for 40%, and NRND water for 24% of hospitalizations from waterborne illnesses. Most direct costs were associated with pathogens found in biofilms. Estimating disease burden by water exposure route helps direct prevention activities. For each exposure route, water management programs are needed to control biofilm-associated pathogen growth; public health programs are needed to prevent biofilm-associated diseases.

Waterborne infectious diseases substantially affect public health in the United States, despite widespread treatment and disinfection of drinking water systems and recreational water venues such as swimming pools and hot tubs. Cases of waterborne infections are estimated at >7.15 million annually in the United States, causing 120,000 hospitalizations and 6,600 deaths (*1*). Pathogens found in biofilms, such as nontuberculous mycobacteria (NTM) and *Legionella* bacteria, are predominant causes of hospitalizations and deaths from waterborne diseases in the United States. Waterborne pathogen exposure routes include swimming, drinking water, bathing, or breathing in aerosolized water.

Before widespread application of drinking water disinfection treatments, cholera and typhoid were major causes of death in the United States. Supplying treated, safe drinking water dramatically reduced the incidence of those diseases; in the past 50 years, outbreaks from large public drinking water systems have occurred less frequently (*2*–*4*), likely because of operational regulations and improvements. However, aging infrastructures and climate change negatively affect drinking water systems (*5*). Furthermore, 43 million US residents are served by private wells or domestic water systems that are not regulated by the Environmental Protection Agency Safe Drinking Water Act, leaving homeowners responsible for maintaining and monitoring water quality in their wells (*6*). During 1971–2008, one third of reported disease outbreaks from drinking water were linked to private wells (*7*). The complexity of water distribution has also increased; 6 million miles of plumbing inside buildings (known as premise plumbing) support drinking water, sanitation, hygiene, cooling, and heating needs in the United States (*8*,*9*). Premise plumbing water quality can be compromised when water is stagnant or disinfectant concentrations are reduced, thereby promoting microbial pathogen growth and biofilm formation (*10*). Exposure to biofilm-related pathogens can occur through contact with, ingestion of, or aerosol inhalation of contaminated water from different sources, such as showerheads, hot tubs, building cooling towers, or decorative fountains.

Filtration and chlorination of water in swimming pools to remove and inactivate pathogens were introduced in the early 1900s. Illnesses caused by recreational water contact still occur, partly because of lack of national standards across the United States for treated recreational water venues, such as pools, hot tubs, and splash pads, and design innovations that have increased venue sizes and complexity and have changed how persons are exposed (e.g., increased hot tub water aerosols).

Swimming in untreated recreational water venues (lakes, rivers, and oceans) can cause outbreaks predominantly linked to norovirus, Shiga toxin–producing *Escherichia coli*, *Cryptosporidium* spp., and *Shigella* spp. (*11*,*12*). Those enteric pathogens can be introduced into untreated recreational water through human feces or vomit, stormwater runoff, sewage or septic system malfunctions, or animal waste and can then be transmitted to persons who ingest the contaminated water.

Annual incidences of illness associated with drinking water in community drinking water systems have been estimated at 4–20 million (*13*–*16*). Illnesses associated with recreational contact with untreated or natural waters have also been estimated (*17*,*18*), and 1 study reported 90 million illnesses annually attributable to recreational water exposure. We previously estimated the overall burden of waterborne diseases in the United States (*1*). In this study, we quantified exposure-specific diseases from 3 water sources—recreational, drinking, and nonrecreational nondrinking (NRND) water—by using a multiplier model of surveillance data from 2014. Our goals were to estimate disease burdens by water exposure route, help guide disease prevention measures, determine key prevention partners, and prioritize limited resources.

## Methods

We apportioned waterborne diseases to recreational, drinking, or NRND water categories as previously described in a structured expert judgment study (*19*) (Appendix Table 1). Recreational water is defined as water used for recreational activities, such as aquatic venues or natural bodies of water. Drinking water is used primarily for drinking but can include other domestic uses, such as washing or showering. Drinking water can come from a public water system, private well, or commercially bottled sources. NRND water is used for purposes other than recreation or consumption, such as agriculture, manufacturing or cooling equipment, or medical treatment; this category also includes backcountry streams and flood waters. Examples of diseases transmitted by NRND water include Legionnaires’ disease associated with a cooling tower, giardiasis transmitted by drinking untreated water from a stream, and *Vibrio* spp. wound infections after wading through flood waters.

We estimated the total number of cases for 17 diseases or syndromes in 2014 in the United States (Appendix): acute otitis externa, campylobacteriosis, cryptosporidiosis, giardiasis, Legionnaires’ disease, NTM infection, norovirus infection, *Pseudomonas* pneumonia, *Pseudomonas* septicemia, salmonellosis (nontyphoidal), Shiga toxin–producing *Escherichia coli* infection with an O157 or non-O157 serogroup, shigellosis, and vibriosis caused by *Vibrio alginolyticus*, *V*. *parahaemolyticus*, *V*. *vulnificus*, and other non–*V*. *cholerae* spp. (*1*) (Appendix Table 2). We divided total waterborne disease estimates previously reported (*1*) by structured expert judgment study estimates (*19*) to produce 2014 burden estimates for recreational, drinking, and NRND water exposure (Appendix Table 3). We quantified direct healthcare costs of treat-and-release emergency department (ED) visits and hospitalizations, measured by insurers and out-of-pocket payments, by using MarketScan data, as previously described (*1*).

We used anonymized administrative surveillance and literature data from 2000–2015 and based all estimates on the 2014 US population (318.6 million persons); at the time of analysis, 2014 was the most recent year for which all data were available. Data sources have been described previously (*1*). We estimated values by using probabilistic multiplier models (Appendix); each model input had an associated uncertainty estimate represented by a distribution of plausible values (Appendix Figure). Uncertainty in final estimates for each type of water exposure was a cumulative effect, incorporating the uncertainties of each model input (*1*,*19*). We obtained count distributions by using 100,000 iterations to generate point estimates of means and corresponding 95% credible intervals (CrIs; the 2.5th–97.5th percentiles of empirical distribution). We generated all-disease totals for each outcome by sampling from the distributions generated for each disease. We used SAS version 9.4 (SAS Institute, https://www.sas.com) and R version 3.5.1 (The R Project for Statistical Computing, https://www.r-project.org) for analyses.

## Results

### Illnesses

In 2014, a total of 5.61 million (95% CrI 2.97–9.00 million) domestically-acquired infectious waterborne illnesses were associated with recreational water, 1.13 million (95% CrI 255,000–3.54 million) with drinking water, and 407,000 (95% CrI 72,800–1.29 million) with NRND water (Table 1). Acute otitis externa caused most illnesses associated with recreational water (79%) and NRND water (27%), and norovirus infection was the leading illness associated with drinking water (53%).

**Table 1:** Number of cases of selected domestically acquired illnesses from different water sources in study estimating waterborne infectious disease burden by exposure route, United States, 2014*

Disease or syndrome	Water exposure route, no. cases (95% CrI)		
	Recreational water†	Drinking water‡	NRND water§
Acute otitis externa	4,430,000 (2,170,000–7,020,000)	126,000 (0–891,000)	111,000 (0–521,000)
Campylobacteriosis	54,900 (0–307,000)	75,400 (40–366,000)	40,900 (0–257,000)
Cryptosporidiosis	211,000 (27,700–718,000)	76,400 (957–362,000)	34,400 (37–177,000)
Giardiasis	204,000 (26,900–552,000)	137,000 (6,070–445,000)	74,100 (41–329,000)
Legionnaires’ disease	1,000 (174–3,810)	5,760 (2,030–9,160)	4,250 (1,360–7,890)
NTM infection	8,630 (0–29,700)	46,400 (17,400–78,200)	13,800 (0–38,200)
Norovirus infection	626,000 (1,930–2,960,000)	604,000 (1,800–2,890,000)	102,000 (2–792,000)
*Pseudomonas* pneumonia	7,600 (996–16,200)	935 (62–4,750)	7,380 (1,330–15,900)
*Pseudomonas* septicemia	417 (23–1,940)	929 (25–3,710)	4,410 (481–11,500)
Salmonellosis, nontyphoidal	14,000 (343–67,500)	57,700 (3,050–214,000)	5,320 (72–31,000)
STEC infection			
O157 serotype	2,300 (188–9,260)	887 (33–4,220)	163 (0–1,050)
Non-O157 serotype	5,780 (0–23,600)	1,360 (0–8,820)	4,300 (0–18,000)
Shigellosis	13,200 (667–60,900)	586 (0–3,920)	3,450 (71–18,800)
*Vibrio* spp. infections	33,500 (184–20,300)	342 (2–210)	759 (3–277)
*V*. *alginolyticus*	12,300 (3,500–24,900)	93 (0–564)	248 (0–2,330)
* V. parahaemolyticus*	20,300 (4,950–38,600)	210 (0–1,900)	277 (0–2,490)
* V. vulnificus*	184 (82–274)	2 (0–18)	3 (0–45)
Other *Vibrio* spp.	610 (0–5,050)	38 (0–270)	231 (0–1,350)
Total illnesses	5,610,000 (2,970,000–9,000,000)	1,130,000 (255,000–3,540,000)	407,000 (72,800–1,290,000)

*Estimates are rounded to 3 significant figures. CrI, credible interval; NRND, nonrecreational nondrinking; NTM, nontuberculous mycobacteria; STEC, Shiga toxin–producing *Escherichia coli.*†Recreational water is used for recreational activities, such as swimming, in treated (e.g., pools, hot tubs, and splash pads) or untreated (e.g., lakes, rivers, and oceans) venues (*19*). ‡Drinking water is used primarily for drinking but can also be used for maintaining hygiene, such as for washing or showering, and can come from public water systems, private wells, or commercially bottled sources (*19*). §NRND water is used for purposes other than recreation or drinking (e.g., for agriculture, industry, or medical procedures) and can come from backcountry streams or flood waters (*19*).

### ED visits

Recreational water exposure was linked to ≈552,000 (95% CrI 320,000–808,000) ED visits, drinking water to 31,600 (95% CrI 4,070–133,000) visits, and NRND water exposure to 17,200 (95% CrI 951–69,400) visits (Table 2). Acute otitis externa caused the most ED visits for each water exposure; 97% of those associated with recreational water, 48% associated with drinking water, and 78% associated with NRND water.

**Table 2:** Number of emergency department visits for selected domestically acquired illnesses from different water sources in study estimating waterborne infectious disease burden by exposure route, United States, 2014*

Disease or syndrome	Water exposure route, no. visits (95% CrI)		
	Recreational water†	Drinking water‡	NRND water§
Acute otitis externa	538,000 (309,000–793,000)	15,300 (0–111,000)	13,500 (0–63,500)
Campylobacteriosis	102 (0–534)	140 (0–630)	76 (0–468)
Cryptosporidiosis	323 (69–732)	117 (2–413)	53 (0–228)
Giardiasis	278 (36–755)	187 (8–615)	102 (0–453)
Legionnaires’ disease	61 (8–234)	349 (96–722)	257 (66–585)
NTM infection	636 (0–2,200)	3,420 (1,240–5,980)	1,020 (0–2,850)
Norovirus infection	12,300 (39–57,200)	11,900 (36–56,100)	2,020 (0–15,700)
*Pseudomonas* pneumonia	139 (18–307)	17 (1–87)	135 (24–301)
*Pseudomonas* septicemia	3 (0–12)	6 (0–26)	28 (1–85)
Salmonellosis, nontyphoidal	35 (0–168)	145 (8–514)	14 (0–78)
STEC infection			
O157 serotype	8 (0–25)	3 (0–12)	0 (0–3)
Non–O157 serotype	2 (0–9)	0 (0–3)	2 (0–7)
Shigellosis	49 (3–236)	2 (0–15)	13 (0–62)
*Vibrio* spp. infections¶	NA	NA	NA
Total visits	552,000 (320,000–808,000)	31,600 (4,070–133,000)	17,200 (951–69,400)

*Estimates are rounded to 3 significant figures. CrI, credible interval; NA, not applicable; NRND, nonrecreational nondrinking; NTM, nontuberculous mycobacteria; STEC, Shiga toxin–producing *Escherichia coli.*†Recreational water is used for recreational activities, such as swimming, in treated (e.g., pools, hot tubs, and splash pads) or untreated (e.g., lakes, rivers, and oceans) venues (*19*). ‡Drinking water is used primarily for drinking but can also be used for maintaining hygiene, such as for washing or showering, and can come from public water systems, private wells, or commercially bottled sources (*19*). §NRND water is used for purposes other than recreation or drinking (e.g., for agriculture, industry, or medical procedures) and can come from backcountry streams or flood waters (*19*). ¶No codes from the International Classification of Diseases, 9th Revision, Clinical Modification, are available for *Vibrio* spp. infections, only a general code for vibriosis and cholera combined. Emergency department visit estimates relied on administrative data that used those codes and are available only for *Vibrio* infections overall.

### Hospitalizations

Recreational water was linked to ≈42,300 (95% CrI 26,500–63,000), drinking water to 47,700 (95% CrI 24,600–72,800), and NRND water to 27,900 (95% CrI 13,200–48,900) hospitalizations (Table 3). Acute otitis externa caused most (52%) hospitalizations associated with recreational water exposure; NTM infections caused most hospitalizations associated with drinking water (73%) and NRND water (37%) exposures.

**Table 3:** Number of hospitalizations for selected domestically acquired illnesses from different water sources in study estimating waterborne infectious disease burden by exposure route, United States, 2014*

Disease or syndrome	Water exposure route, no. hospitalizations (95% CrI)		
	Recreational water†	Drinking water‡	NRND water§
Acute otitis externa	22,000 (12,700–32,400)	628 (0–4,590)	553 (0–2,630)
Campylobacteriosis	690 (0–3,700)	947 (0–4,410)	513 (0–3,190)
Cryptosporidiosis	734 (56–2,560)	265 (3–1,260)	119 (0–625)
Giardiasis	540 (71–1,470)	362 (16–1,180)	196 (0–870)
Legionnaires’ disease	985 (171–3,740)	5,650 (1,990–8,990)	4,170 (1,340–7,740)
NTM infection	6,440 (0–22,200)	34,600 (13,000–58,000)	10,300 (0–28,400)
Norovirus infection	2,240 (7–10,300)	2,170 (7–10,100)	367 (0–2,840)
*Pseudomonas* pneumonia	7,390 (970–15,700)	909 (60–4,620)	7,170 (1,290–15,400)
*Pseudomonas* septicemia	405 (22–1,890)	903 (24–3,600)	4,290 (468–11,100)
Salmonellosis, nontyphoidal	275 (6–1,360)	1,140 (56–4,400)	105 (1–617)
STEC infection			
O157 serotype	95 (8–364)	36 (1–165)	7 (0–42)
Non-O157 serotype	38 (0–165)	9 (0–61)	28 (0–126)
Shigellosis	188 (8–886)	8 (0–56)	49 (0–264)
*Vibrio* spp. infections	243 (3–157)	2 (0–1)	5 (0–3)
* V. alginolyticus*	25 (7–57)	0 (0–1)	0 (0–5)
* V. parahaemolyticus*	58 (14–111)	0 (0–5)	0 (0–7)
* V. vulnificus*	157 (69–239)	1 (0–15)	3 (0–38)
Other *Vibrio* spp.	3 (0–27)	0 (0–2)	1 (0–7)
Total hospitalizations	42,300 (26,500–63,000)	47,700 (24,600–72,800)	27,900 (13,200–48,900)

*Estimates are rounded to 3 significant figures. CrI, credible interval; NRND, nonrecreational nondrinking; NTM, notuberculous mycobacteria; STEC, Shiga toxin–producing *Escherichia* *coli*. †Recreational water is used for recreational activities, such as swimming, in treated (e.g., pools, hot tubs, and splash pads) or untreated (e.g., lakes, rivers, and oceans) venues (*19*). ‡Drinking water is used primarily for drinking but can also be used for maintaining hygiene, such as for washing or showering, and can come from public water systems, private wells, or commercially bottled water sources (*19*). §NRND water is used for purposes other than recreation or drinking (e.g., agriculture, industry, or medical procedures) and can come from backcountry streams or flood waters (*19*).

### Deaths

Recreational water exposure was linked to ≈1,290 (95% CrI 591–2,520) deaths, drinking water to 3,300 (95% CrI 1,630–5,180) deaths, and NRND water exposure to 2,040 (95% CrI 909–3,690) deaths (Table 4). NTM infections caused most deaths for each water exposure type; 37% of NTM-related deaths were associated with recreational water, 78% with drinking water, and 37% with NRND water.

**Table 4:** Number of deaths from selected illnesses domestically acquired from different water sources in study estimating waterborne infectious disease burden by exposure route, United States, 2014*

Disease or syndrome	Water exposure route, no. deaths (95% CrI)		
	Recreational water†	Drinking water‡	NRND water§
Acute otitis externa	208 (98–352)	6 (0–41)	5 (0–24)
Campylobacteriosis	9 (0–65)	12 (0–81)	6 (0–49)
Cryptosporidiosis	16 (0–96)	6 (0–46)	3 (0–22)
Giardiasis	0 (0–3)	0 (0–2)	0 (0–2)
Legionnaires’ disease	91 (15–347)	520 (180–858)	384 (122–727)
NTM infection	476 (0–1,650)	2,560 (950–4,370)	763 (0–2,110)
Norovirus infection	25 (0–116)	25 (0–114)	4 (0–32)
*Pseudomonas* pneumonia	349 (44–795)	43 (3–213)	339 (58–779)
*Pseudomonas* septicemia	50 (3–236)	112 (3–449)	532 (58–1,390)
Salmonellosis, nontyphoidal	4 (0–24)	18 (0–79)	2 (0–11)
STEC infections			
O157 serotype	1 (0–12)	0 (0–5)	0 (0–0)
Non-O157 serotype	0 (0–6)	0 (0–1)	0 (0–4)
Shigellosis	0 (0–7)	0 (0–0)	0 (0–2)
*Vibrio* spp. infections	58 (0–53)	0 (0–0)	1 (0–0)
* V. alginolyticus*	1 (0–4)	0 (0–0)	0 (0–0)
* V. parahaemolyticus*	4 (0–9)	0 (0–0)	0 (0–0)
* V. vulnificus*	53 (21–84)	0 (0–5)	0 (0–13)
Other *Vibrio* spp.	0 (0–2)	0 (0–0)	0 (0–0)
Total deaths	1,290 (591–2,520)	3,300 (1,630–5,180)	2,040 (909–3,690)

*Estimates are rounded to 3 significant figures. CrI, credible interval; NRND, nonrecreational nondrinking; NTM, nontuberculous mycobacteria; STEC, Shiga toxin–producing *Escherichia coli.*†Recreational water is used for recreational activities (e.g., swimming) in treated (e.g., pools, hot tubs, and splash pads) or untreated (e.g., lakes, rivers, and oceans) venues (*19*). ‡Drinking water is used primarily for drinking but can also be used for hygiene activities, like washing or showering, and can come from a public water system, a private well, or commercially bottled sources (*19*). §NRND water is used for purposes other than recreation or drinking (e.g., for agriculture, industry, medical treatment, backcountry streams, or flood waters) (*19*).

### Direct healthcare costs for ED visits and hospitalizations

Illnesses associated with drinking water represented the largest portion (42%) of combined costs, totaling $1.39 billion (95% CrI $364.00 million–$4.81 billion) (Figure; Appendix Tables 4, 5). Illnesses associated with recreational water exposures represented 32%, totaling $1.07 billion (95% CrI $439.00 million–$2.67 billion) of combined costs; illnesses associated with NRND exposures made up 26%, totaling $871.00 million (95% CrI $240.00 million–$2.64 billion) of combined costs. Four infection types accounted for 89% of $3.33 billion total costs; NTM infection was responsible for 46%, acute otitis externa for 17%, *Pseudomonas* pneumonia for 14%, and Legionnaires’ disease for 12% of combined costs (Appendix Tables 4, 5).

**Figure:**
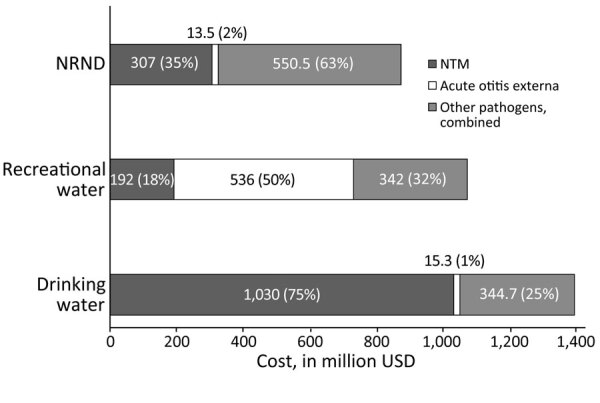
Direct healthcare costs for emergency department visits and hospitalizations in study estimating waterborne infectious disease burden by exposure route, United States, 2014. Estimated combined costs are shown in US dollars for selected domestically acquired waterborne illnesses for each exposure route. Estimates were rounded to 3 significant figures. NRND water is used for purposes other than recreation or drinking (e.g., agriculture, industry, medical treatment, backcountry streams, or flood waters); recreational water is used for recreational activities in treated (e.g., pools, hot tubs, and splash pads) or untreated (e.g., lakes, rivers, and oceans) venues; drinking water is used for drinking, washing, or showering and can come from a public water system, private well, or commercially bottled sources (*19*). NRND, nonrecreational nondrinking; NTM, nontuberculous mycobacteria.

## Discussion

Of the estimated 7.15 million infectious waterborne illnesses in 2014 in the United States, 78% of illnesses were attributed to recreational water, 16% to drinking water, and 6% to NRND water. Drinking water exposure caused 40% of hospitalizations and 50% of deaths, recreational water exposure 36% of hospitalizations and 20% of deaths, and NRND exposure 24% of hospitalizations and 30% of deaths. ED visits and hospitalizations from exposure to drinking or recreational water exposure cost >$2 billion annually.

Acute otitis externa, which can be acutely painful and cause itchiness, drainage, and swelling, was the most common recreational water–associated illness. Risks for acute otitis externa have been correlated with water quality measures, levels of *Pseudomonas* and other pathogens in water, and increased bather load in recreational water venues in some studies but not others (*20*–*23*). Water can displace the protective coating of cerumen (ear wax) in the ear canal, leaving the outer ear more vulnerable to infection by endogenous flora. Whether otitis externa is caused by endogenous flora or contamination introduced by water remains unclear. In the structured expert judgment study (*19*), experts were asked to consider this question while estimating the proportion of otitis externa transmitted through water versus other pathways. They estimated 81% (95% uncertainty interval 67%–95%) of *Pseudomonas* otitis externa was transmitted through water (*19*). In response to the absence of national standards for recreational water venues, the Centers for Disease Control and Prevention (CDC) participated in the development and updating of the Model Aquatic Health Code (MAHC) (*24*). For treated water venues, the MAHC includes guidance for health authorities and aquatics sector to minimize risks for acute otitis externa and other illnesses and injuries (*24*,*25*). For both treated and untreated venues, risk for acute otitis externa can be minimized by keeping ears as dry as possible when swimming and ensuring ears are dry after swimming. Recreational swimmers and parents of young swimmers can educate themselves about steps to minimize the risks for infection from enteric pathogens at trusted sites, such as the CDC’s healthy swimming webpage (*26*). Signage at treated and untreated recreational water venues (*12*) and adoption of CDC’s MAHC for treated public recreational water venues can further improve prevention. The number of illnesses and ED visits for acute otitis externa caused by drinking water exposure might seem counterintuitive, but drinking water was defined as water used for drinking, bathing, or showering.

*Cryptosporidium* and *Legionella* spp. infections in humans were identified in the late 1970s, and outbreaks associated with those pathogens in treated recreational water venues were identified soon after. In subsequent decades, reported incidences of treated recreational water–associated outbreaks increased substantially; *Cryptosporidium*, *Legionella*, and *Pseudomonas* spp. were the predominant etiologies (*27*). *P.* *aeruginosa* is a major cause of acute otitis externa and folliculitis or hot tub rash in addition to pneumonia and septicemia (*28*). Infectious *Cryptosporidium* sp. oocysts have tough outer shells that provide extreme chlorine tolerance, whereas *Legionella* and *Pseudomonas* spp. thrive in biofilms, where they are protected from chlorine inactivation.

Reported infectious disease outbreaks associated with public drinking water systems have decreased since the 1970s, likely because of federal management and treatment regulations that address enteric pathogens (*3*). However, outbreaks associated with individual water systems, such as private wells, and premise plumbing deficiencies have not decreased (*3*). Our analysis affirms that most hospitalizations and deaths associated with drinking water exposure were linked to biofilm-associated pathogens, not to pathogens causing enteric disease. *Legionella* bacteria are biofilm-associated pathogens and have become the most frequently reported cause of disease outbreaks associated with drinking water. Our analysis revealed that NTM infections were the leading cause of drinking water–associated illnesses, ED visits, hospitalizations, and deaths; NTM infections were the most common cause of death associated with all 3 water exposure routes. However, NTM infections are not nationally notifiable diseases; thus, cases are not consistently reported to public health authorities and outbreaks might remain undetected. Improving NTM illness reporting could lead to greater outbreak detection and inform disease prevention strategies for water systems. The Council of State and Territorial Epidemiologists has a standardized case definition for extrapulmonary NTM infections (opportunistic infections of wounds, soft tissue, or joints) to increase reportability, ensure consistency in reporting, and help identify outbreaks (*29*). Thus far, the state of Oregon has adopted this definition for their surveillance program (*30*), and other sites are piloting NTM surveillance programs (*31*).

NRND water includes water used for agriculture, such as for irrigation or livestock; industry, such as manufacturing or cooling equipment; medical procedures, such as medical devices, washing surgical tools and equipment, and hydrotherapy; and backcountry streams or floodwaters (*19*). NRND water made up 87% of the 322 billion gallons of water used each day in the United States in 2015; of that volume, 2 billion gallons were used for livestock, 118 billion gallons for irrigation, 7.55 billion gallons for aquaculture, and 133 billion gallons for thermoelectric power (*6*). NRND water is associated with a small portion of reported waterborne illnesses but is responsible for one quarter of waterborne disease–associated hospitalizations and one third of associated deaths.

Waterborne pathogens, such as NTM, *Pseudomonas* spp., and *Legionella* spp., occur naturally in freshwater and can colonize other environments, particularly large complex equipment that is not properly maintained. Those pathogens are aerosolized in water droplets produced during use, which can be inhaled. Water management programs are recommended to minimize growth and spread of pathogens in engineered systems (*32**–**34*). Resources can be found on the CDC Environmental Health Services safe water program webpage (*35*), guide for developing a water management program to reduce *Legionella* growth and spread in buildings (*36*), *Legionella* control toolkit (*37*), and guide for reducing risk from water (*38*). Water management programs in healthcare facilities are also critical for protecting vulnerable patient populations, staff, and visitors and might require specific considerations for a wide range of biofilm-associated pathogens, including NTM, *Pseudomonas*, and *Legionella* spp. (*39*–*42*). Interdisciplinary collaboration is needed for planning future management of complex water systems and investigating illnesses and outbreaks.

Reducing risks for illness from biofilms in water systems is difficult. Biofilms can persist despite standard water treatment processes. Biofilms are complex ecosystems; control is complicated by structural issues (e.g., pipe characteristics), system operational issues (e.g., water age, temperature, and residual disinfectants), and water end-user behaviors. Changes designed to reduce prevalence of 1 microbial constituent can sometimes produce unintended consequences, such as proliferation of NTM, if a comprehensive approach to biofilm control is not considered (*43*). NTM are persistent pathogens; despite standard treatment, NTM species have been recovered from surface water treatment plants, biofilms in sand filters, ozonated water, biofilms from granular activated carbon filters, and activated carbon-filtered water. Even municipal systems that incorporate full treatment chains, including 2 filtration and 2 disinfection stages, might not effectively prevent NTM growth (*44*). Because climate change continues to create extreme weather events and increase water temperatures, more stress will be placed on aging water infrastructure; biofilm-associated pathogens will continue to proliferate (*5*), highlighting the need for biofilm-focused control programs.

Prevention of biofilm-associated organisms is an emerging field, and more scientific evidence is needed to determine best practices for public water systems and premise plumbing and identify effective prevention strategies for homeowners and building managers. Persons can reduce their risk for illness from a biofilm-associated pathogen by flushing rarely used faucets and showerheads, flushing water heaters, and maintaining specific water heater temperatures. Resources for risk reduction can be found on CDC’s Preventing Waterborne Germs at Home webpage (*45*).

Most previous waterborne infectious disease estimates are not directly comparable with results from this study because of differing methods used to generate estimates. Many studies have attempted to estimate a burden for 1 disease or aspect of drinking or recreational water exposure. Two studies from a workshop convened by CDC and the Environmental Protection Agency used 2 different methods to estimate the number of cases of acute gastrointestinal illness in public drinking water systems only. Those studies estimated 4–16 million acute gastrointestinal illness cases per year (*13*,*16*). A 2008 study estimated all illnesses associated with public drinking water systems at 19 million cases per year (*14*). Previous publications have used cohort studies to estimate gastrointestinal illness from water recreational activities and percentages of economic burden from recreational water exposure (*17*,*46*). In Canada, rates for waterborne disease per 100,000 persons were estimated by using literature, clinical input, and administrative data (*47*). Our estimates differ from previous work because we focused on specific pathogens, including those causing nongastrointestinal diseases, and we provide estimates for recreational water, drinking water, and NRND water exposure routes.

The first limitation of our study is that the estimates relied on a series of multipliers. Although we attempted to account for uncertainty in the multiplier estimates, biased multipliers will yield biased estimates. We used attribution estimates for proportions of disease caused by each water exposure route, derived from a structured expert judgment study. Structured expert judgment is used when data are sparse and relies on expert opinion. Statistical techniques are used to combine expert opinions into a single estimate with an uncertainty interval. Various weighting techniques can be used to increase accuracy or informativeness (narrowness of the uncertainty interval), but tradeoffs between accuracy and informativeness occur. For some individual estimates, including norovirus infection, NTM infection, and acute otitis externa, uncertainty intervals were wide, reflecting uncertainty about the proportion of disease transmitted via water. Second, as with previous waterborne disease burden estimates (*1*), we included estimates of 17 infectious diseases that had adequate data available. Other waterborne infectious diseases were excluded, such as *Pseudomonas* folliculitis or viral gastroenteritis not caused by norovirus. We also excluded noninfectious diseases caused by exposure to contaminated water, such as from lead or harmful algae-derived toxins, and did not evaluate long-term chronic effects of infectious or noninfectious diseases. In addition, we excluded foodborne illnesses, such as salmonellosis from lettuce or norovirus disease from shellfish, where contaminated water might have been responsible. Third, this study used data from hospital and ED billing databases. Billing records are not medical records and might reflect diagnoses that result in reimbursement from insurance companies rather than true assessments by clinicians. Fourth, those billing databases use International Classification of Diseases, 9th Revision, Clinical Modification, coding to classify diagnoses; some illnesses might not perfectly match existing codes. Fifth, we provide estimates for 2014, and illness burdens, healthcare costs, and clinical practices have likely changed since then. Sixth, we assumed that severity of illness was independent of exposure route. For example, if salmonellosis was more severe when transmitted by drinking water than by recreational water, the multipliers used in calculating estimates did not account for this. Seventh, many diseases in this analysis might be more severe in persons with compromised immunity, but we did not attempt to apportion the burden of illness between waterborne illnesses and immune status. Finally, reliably distinguishing between illnesses linked to premise plumbing water exposure and distribution system exposure was difficult, which was reflected in 95% CrI widths for drinking water and NRND estimates for some pathogens, including NTM. More research is needed in this area because federal regulations for distribution systems differ from building codes and state and local laws that regulate premise plumbing.

In conclusion, quantifying the burden of illness linked to drinking, recreational, and NRND water is an essential factor for addressing biofilm-associated pathogens, because prevention measures and vital partners differ for each exposure route. Estimating disease burden by water exposure route will help direct prevention activities and prioritize limited resources. Measures for preventing illnesses from recreational water use, such as those in the MAHC for treated recreational water venues, could reduce disease burden associated with recreational water exposure. Incorporating water management programs and control measures for systems that are common sources of biofilm pathogen exposure into building and public health codes is crucial for reducing risks for biofilm-associated pathogen exposure in drinking water and NRND water systems (*34*). In addition, improved surveillance and reporting of biofilm-associated illnesses could lead to the timely detection and investigation of outbreaks and inform disease prevention activities. Interdisciplinary partnerships among public health agencies and industries that use NRND water are pivotal to reduce incidences of waterborne diseases. For drinking water and NRND water systems, addressing factors that promote biofilm growth (stagnation, temperature, or lack of disinfection) could reduce growth of biofilm-associated pathogens. Facilities that serve persons at increased risk for infections, such as healthcare facilities, should review water system design and operations to decrease risks from biofilm-associated pathogens. Additional research on the roles of pipe material, water chemistry, and optimal water distribution system management could provide insights into improving prevention actions. As climate change continues to place increased stress on water systems, improved prevention and maintenance strategies should be developed to prevent illness from all types of water exposure.

Appendix Additional information for estimating waterborne infectious disease burden by exposure route, United States, 2014.
