# Supplementary material for: Estimating Waterborne Infectious Disease Burden by Exposure Route, United States, 2014
Source: Emerg Infect Dis. 2023 Jul;29(7):1357–66. doi: 10.3201/eid2907.230231 (PMC10310388; doi:10.3201/eid2907.230231)
Supplement: Appendix — Additional information for estimating waterborne infectious disease burden by exposure route, United States, 2014. [file 23-0231-Techapp-s1.pdf]

Article DOI: https://doi.org/10.3201/eid2907.230231

EID cannot ensure accessibility for supplementary materials supplied by authors. Readers who have difficulty accessing supplementary content should contact the authors for assistance.

Estimating Waterborne Infectious Disease Burden by Exposure Route, United States, 2014

Appendix

Additional Methods

Definitions

Waterborne transmission was defined as consumption of, direct contact with, or aerosolized inhalation of water. We used the exposure route definitions for recreational, drinking, and nonrecreational nondrinking (NRND) water as previously described (*1*) (Appendix Table 1). For all transmission pathways, the point of attribution was defined as the point of exposure (i.e., the event during which a person was exposed to the pathogen) (*1*).

Acute otitis externa was defined as otitis externa without concurrent otitis media. No national case surveillance system for otitis externa exists; therefore, we used administrative data and case definitions from the International Classification of Diseases, 9th Edition, Clinical Modification (ICD-9-CM) codes (*2*). We used ICD-9-CM codes 380.10 (infective otitis externa, unspecified), 380.12 (acute swimmers' ear), and 380.14 (malignant otitis externa). Because distinguishing otitis externa from otitis media is difficult and a conservative estimate was desired, all visits with a concurrent diagnosis of ICD-9-CM code 381 (nonsuppurative otitis media and eustachian tube disorders) or 382 (suppurative and unspecified otitis media) were excluded. Acute otitis externa can be caused by many pathogens, which makes calculating the burden more complicated. We conducted a literature search to estimate the proportion of acute otitis externa caused by each causative agent. The literature in this area was sparse, but 1 study estimated 50% of otitis externa was caused by *Pseudomonas* sp., 25% by staphylococci, and the remainder by other pathogens (*3*). For estimation purposes, we asked experts who participated in

the structured expert judgment study to estimate incidences for acute otitis externa caused by *Pseudomonas* sp. and by S. *aureus* (*1*). We then combined the estimates and uncertainty distributions generated by the experts for each pathogen into a single 100,000-observation dataset with a weight ratio of 2:1 *Pseudomonas*:staphylococci.

Modeling Methods and Probability Distributions

We used a probability modeling approach; for each disease or syndrome, a probability distribution for each measure of overall burden (e.g., number of illnesses, hospitalizations) was multiplied by a probability distribution of the proportion transmitted through each pathway. This study built on research described previously (*1,4*) and used datasets from both previous projects. Datasets from Collier et al. (*4*) were used for estimating the burden of domestically-acquired waterborne diseases by disease or syndrome for all 3 exposure pathways. Datasets from Beshearse et al. (*1*) were used for estimating proportions of each disease or syndrome transmitted through each of the 3 water exposure pathways. Both datasets contained 100,000 observations for each of the 17 pathogens in the estimates. Multiplying each disease, outcome, and pathway 100,000 times resulted in a dataset with 100,000 estimates of the number of illnesses, hospitalizations, or deaths for a given disease/syndrome within each exposure pathway. The numbers reported in the tables are descriptions of the means and 2.5th and 97.5th percentiles in the datasets. The point estimates and credible intervals reported in this study could not be directly derived from the numbers reported previously in tables (*1,4*) through simple multiplication, because the distributions of the datasets were not symmetric. The total numbers of cases of Legionnaires' disease from drinking water are shown as an example of the calculations used in this study (Appendix Figure). Note that simply multiplying 11,000 × 52% equals 5,720, not 5,760. This process was repeated for each water exposure pathway, outcome (illness, emergency department visit, hospitalization, death, emergency department visit cost, or hospitalization cost), and disease or syndrome to create the estimates presented in this study.

References

1. Beshearse E, Bruce BB, Nane GF, Cooke RM, Aspinall W, Hald T, et al. Attribution of illnesses transmitted by food and water to comprehensive transmission pathways using structured expert judgment, United States. Emerg Infect Dis. 2021;27:182–95. PubMed https://doi.org/10.3201/eid2701.200316

2. Centers for Disease Control and Prevention. International classification of diseases, ninth revision, clinical modification (ICD-9-CM) [cited 2023 Apr 4]. https://www.cdc.gov/nchs/icd/icd9cm.htm

3. Roland PS, Stroman DW. Microbiology of acute otitis externa. Laryngoscope. 2002;112:1166–77. PubMed https://doi.org/10.1097/00005537-200207000-00005

4. Collier SA, Deng L, Adam EA, Benedict KM, Beshearse EM, Blackstock AJ, et al. Estimate of burden and direct healthcare cost of infectious waterborne disease in the United States. Emerg Infect Dis. 2021;27:140–9. PubMed https://doi.org/10.3201/eid2701.190676

Appendix Table 1. Transmission pathway definitions*

Exposure pathway	Description
Recreational water, treated or untreated	Water used for recreational activities, such as aquatic facilities or natural bodies of water. Water can be treated or untreated. Treated water has undergone a systematic disinfection process (e.g., chlorination and filtration) with the goal of maintaining good microbiologic quality for recreation; untreated water has not undergone a disinfection or treatment process to maintain good microbiologic quality for recreation (e.g., lakes, rivers, oceans, and reservoirs).
Drinking water	Water used primarily for drinking but including other domestic uses, such as washing or showering. Water can come from a public water system, private well, or commercially bottled water sources.
Nonrecreational nondrinking water	Water used for purposes other than recreation or drinking (e.g., agriculture, industry, medical procedures). Water can also come from backcountry streams or flood waters. Agricultural water includes water used to grow fresh produce and sustain livestock. Industrial water includes water used during manufacturing or for cooling equipment. Medical water includes any water used within medical devices, for washing surgical tools and equipment, and for hydrotherapy. This subcategory does not include disease transmission that can be accounted for by another major pathway, such as food or animals.

*Adapted from (*1*).

Appendix Table 2. Estimated total number of cases of selected domestically-acquired waterborne infectious diseases in 2014, United States*

Disease or syndrome	Confirmed cases	Multipliers		Total no. cases (95% CrI)	International travel, %	% Waterborne (95% CrI)	Domestically-acquired disease, no. (95% CrI)
		Underreported	Underdiagnosed				
Campylobacteriosis	54,000	1.0	28.3	1,540,000 (597,000–3,250,000)	14.4	13 (1–31)	171,000 (13,900–586,000)
Cryptosporidiosis	8,450	1.0	97.3	823,000 (243,000–2,160,000)	9.9	43 (17–73)	322,000 (61,700–993,000)
Giardiasis	17,900	1.30	45.9	1,070,000 (727,000–1,560,000)	12.3	44 (16–78)	415,000 (140,000–816,000)
Legionnaires' disease	5,030	1.0	2.3	11,400 (8,920–13,600)	1.0	97 (67–100)	11,000 (7,430–13,300)
NTM infection	25,700	1.0	3.8	97,000 (75,700–122,000)	1.0	72 (39–94)	68,900 (35,800–100,000)
Norovirus	NA	1.0	NA	21,800,000 (12,100,000–36,000,000)	1.1	6 (0–25)	1,330,000 (5,310–5,510,000)
Otitis externa†	1,720,000	1.0	3.4	5,980,000 (3,200,000–8,880,000)	1.3	79 (67–95)†	4,670,000 (2,350,000–7,290,000)
Pseudomonas pneumonia	15,800	1.0	2.0	31,700 (19,300–46,000)	1.0	51 (14–80)	15,900 (4,240–29,000)
Pseudomonas septicemia	13,000	1.0	2.0	26,100 (16,700–35,900)	1.0	22 (3–53)	5,760 (743–14,400)
Salmonellosis, nontyphoidal	46,400	1.0	29.1	1,350,000 (733,000–2,450,000)	9.7	6 (0–22)	77,000 (5,640–277,000)
STEC infections							
O157 serotype	3,530	1.0	18.2	64,200 (13,000–188,000)	4.0	5 (1–13)	3,360 (336–12,900)
Non-O157 serotype	4,550	1.0	48.1	219,000 (80,000–493,000)	15.3	6 (0–17)	11,400 (0–43,900)
Shigellosis	13,600	1.0	33.1	449,000 (97,800–1,350,000)	7.8	4 (1–21)	17,300 (1,080–77,500)
Vibrio spp. infections	1,230	NA	NA	172,000 (126,000–231,000)	NA	NA	34,600 (17,600–56,900)
V. alginolyticus	234	1.1	142.8	36,700 (23,600–54,800)	6.5	37 (13–71)	12,700 (4,100–25,400)
V. parahaemolyticus	593	1.1	141.6	92,400 (55,000–144,000)	6.7	24 (7–38)	20,800 (6,000–39,000)
V. vulnificus	133	1.1	1.7	249 (178–340)	1.5	77 (40–91)	188(93–277)
Other *Vibrio* spp.	271	1.1	142.8	42,600 (25,500–66,500)	14.4	2 (0–23)	879 (3–8,490)
Total no. case	NA	NA	NA	33,600,000 (23,500,000–48,000,000)	NA	NA	7,150,000 (3,880,000–12,000,000)

*Estimates rounded to 3 significant figures. Data were adapted from (*4*). CrI, credible interval; NA, not applicable; NTM, nontuberculous mycobacteria; STEC, Shiga toxin–producing *Escherichia coli.*
†Combines the waterborne source attribution *(1)* for otitis externa caused by *Pseudomonas* spp. (81%) and *Staphylococcus aureus* (75%) in a ratio of 2:1.

Appendix Table 3. Source attribution results for waterborne transmission subpathways from structured expert judgment study*

Pathogen name	Mean % (95% uncertainty interval) Recreational water†	Drinking water‡	NRND water§
Campylobacter spp.	32 (0–97)	44 (0–99)	24 (0–99)
Cryptosporidium spp.	66 (21–96)	24 (0–68)	11 (0–41)
Giardia spp.	49 (9–93)	33 (2–82)	18 (0–67)
Legionella spp.	9 (2–35)	52 (19–78)	39 (13–69)
NTM spp.	13 (0–43)	67 (33–93)	20 (0–51)
Norovirus	47 (8–90)	45 (6–86)	8 (0–42)
Pseudomonas spp., otitis externa	95 (75–100)	3 (0–21)	2 (0–11)
Pseudomonas spp., pneumonia	48 (17–74)	6 (1–33)	46 (18–76)
Pseudomonas spp., septicemia	7 (2–37)	16 (1–50)	77 (37–94)
Salmonella enterica, nontyphoidal	18 (2–53)	75 (37–93)	7 (0–26)
Staphylococcus aureus	91 (50–100)	5 (0–29)	4 (0–43)
STEC O157	69 (33–94)	26 (3–60)	5 (0–28)
STEC non-O157	51 (18–77)	12 (0–43)	38 (12–69)
Shigella spp.	77 (41–95)	3 (0–25)	20 (3–50)
Vibrio alginolyticus AGI	97 (66–100)	1 (0–6)	2 (0–21)
V. alginolyticus, non-AGI	96 (49–100)	2 (0–36)	3 (0–47)
V. cholerae nontoxigenic AGI	96 (56–100)	2 (0–11)	2 (0–22)
V. cholerae nontoxigenic, non-AGI	96 (50–100)	2 (0–14)	3 (0–43)
V. parahaemolyticus	98 (62–100)	1 (0–10)	1 (0–13)
V. parahaemolyticus, non-AGI	97 (50–100)	2 (0–35)	2 (0–37)
V. vulnificus¶	98 (66–100)	1 (0–9)	2 (0–24)
V. vulnificus, non-AGI	96 (49–100)	2 (0–37)	2 (0–43)
Vibrio spp., other AGI	69 (0–100)	4 (0–69)	27 (0–100)
Vibrio spp, other non-AGI	70 (0–100)	4 (0–69)	26 (0–100)

*Data were adapted from a structured expert judgment study (*1*). AGI, acute gastrointestinal disease; NRND, nonrecreational nondrinking; NTM, nontuberculous mycobacteria; STEC, Shiga toxin–producing *Escherichia coli*.
†Recreational water is used for recreational activities in treated (e.g., pools, hot tubs, and splash pads) or untreated (e.g., lakes, rivers, and oceans) venues.
‡Drinking water is used primarily for drinking but can also be used for hygienic activities, such as washing or showering, and can come from a public water system, private well, or commercially bottled sources.
§NRND water is used for purposes other than recreation or drinking (e.g., agriculture, industry, medical treatment, backcountry streams, or flood waters).
¶Initial clinical manifestations of interest were bacteremia and wound infections.

Appendix Table 4. Estimated cost of ED visits and hospitalizations for selected domestically-acquired infectious waterborne illnesses according to exposure route, United States. 2014*

	Estimated cost, US dollars					
	ED visits			Hospitalizations		
Disease or syndrome	Recreational water (95% CrI)	Drinking water (95% CrI)	NRND water (95% CrI)	Recreational water (95% CrI)	Drinking water (95% CrI)	NRND water (95% CrI)
Acute otitis externa	266,000,000 (56,400,000–805,000,000)	7,560,000 (2–48,900,000)	6,680,000 (2–35,500,000)	270,000,000 (63,100,000–992,000,000)	7,710,000 (2–51,900,000)	6,780,000 (2–38,400,000)
Campylobacteriosis	175,000 (1–1,200,000)	240,000 (79–1,460,000)	130,000 (2–922,000)	9,720,000 (86–55,800,000)	13,300,000 (5,230–68,900,000)	6,970,000 (100–44,000,000)
Cryptosporidiosis	631,000 (41,000–2,420,000)	229,000 (1,860–1,180,000)	103,000 (94–569,000)	11,800,000 (571,000–55,200,000)	4,250,000 (28,200–24,100,000)	1,910,000 (1,490–11,500,000)
Giardiasis	451,000 (22,500–2,050,000)	302,000 (6,420–1,510,000)	164,000 (63–987,000)	11,800,000 (794,000–55,200,000)	7,870,000 (196,000–39,800,000)	4,240,000 (1,630–25,200,000)
Legionnaires' disease	41,900 (4,160–174,000)	241,000 (48,400–646,000)	178,000 (33,200–505,000)	36,500,000 (2,460,000–192,000,000)	210,000,000 (29,100,000–925,000,000)	155,000,000 (20,000,000–700,000,000)
NTM infection	1,030,000 (0–5,670,000)	5,510,000 (336,000–23,800,000)	1,640,000 (0–8,590,000)	191,000,000 (0–959,000,000)	1,030,000,000 (142,000,000–4,380,000,000)	306,000,000 (0–1,480,000,000)
Norovirus infection	14,020,000	13,600,000	2,303,000	13,600,000	13,200,000	2,230,000
Pseudomonas pneumonia	119,000 (5,040–622,000)	14,600 (338–82,400)	115,000 (5,770–607,000)	216,000,000 (14,300,000–958,000,000)	26,800,000 (930,000–142,000,000)	210,000,000 (17,500,000–935,000,000)
Pseudomonas septicemia	2,400 (23–14,200)	5,440 (27–34,800)	25,600 (478–144,000)	15,400,000 (394,000–86,000,000)	34,500,000 (459,000–196,000,000)	164,000,000 (7,520,000–806,000,000)
Salmonellosis, nontyphoidal	43,400 (513–261,000)	180,000 (4,350–945,000)	16,700 (110–112,000)	4,060,000 (60,800–22,700,000)	16,900,000 (496,000–83,600,000)	1,550,000 (12,700–9,930,000)
STEC infection						
O157 serotype	8,910 (299–36,700)	3,480 (60–17,200)	623 (0–4,100)	1,830,000 (72,900–10,100,000)	707,000 (14,000–4,170,000)	129,000 (0–924,000)
non–O157 serotype	2,230 (0–11,800)	525 (0–4,040)	1,650 (0–8,850)	889,000 (0–5,600,000)	209,000 (0–1,620,000)	661,000 (0–4,260,000)
Shigellosis	46,600 (1,120–264,000)	1,990 (0–14,500)	12,300 (143–74,000)	2,610,000 (69,000–14,800,000)	116,000 (0–813,000)	683,000 (8,520–4,080,000)
Vibrio spp. infection	75,500 (6,170–270,000)	694 (0–4,900)	1,510 (0–14,400)	3,910,000 (721,000–10,600,000)	34,700 (9–289,000)	77,800 (10–808,000)
Total Cost	282,000,000 (73,100,000–823,000,000)	27,800,000 (15,100,000–74,600,000)	11,400,000 (2,870,000–40,900,000)	789,000,000 (272,000,000–2,250,000,000)	1,360,000,000 (340,000,000–4,780,000,000)	860,000,000 (232,000,000–2,630,000,000)

*Estimates are rounded to 3 significant figures. Recreational water is used for recreational activities in treated (e.g., pools, hot tubs, and splash pads) or untreated (e.g., lakes, rivers, and oceans) venues; drinking water is used primarily for drinking but can also be used for hygienic activities, such as washing or showering, and can come from a public water system, private well, or commercially bottled sources; NRND water is used for purposes other than recreation or drinking (e.g., agriculture, industry, medical treatment, backcountry streams, or flood waters) (*1*). CrI, credible interval; ED, emergency department; NRND, nonrecreational nondrinking; NTM, nontuberculous mycobacterial; STEC, Shiga toxin-producing *Escherichia coli*.

Appendix Table 5. Estimated total cost of emergency department visits plus hospitalizations for selected domestically-acquired waterborne infectious diseases according to water exposure route, United States, 2014*

	Estimated total costs, US dollars		
Disease or syndrome	Recreational water (95% CrI)	Drinking water (95% CrI)	NRND water (95% CrI)
Acute otitis externa	536,000,000 (173,000,000–1,490,000,000)	15,300,000 (4–102,000,000)	13,500,000 (4–69,600,000)
Campylobacteriosis	9,900,000 (93–56,200,000)	13,500,000 (5,830–69,400,000)	7,100,000 (104–44,300,000)
Cryptosporidiosis	12,400,000 (906,000–56,000,000)	4,480,000 (37,700–24,400,000)	2,010,000 (1,710–11,800,000)
Giardiasis	12,300,000 (914,000–55,700,000)	8,180,000 (217,000–40,400,000)	4,410,000 (1,760–25,500,000)
Legionnaires' disease	36,600,000 (2,470,000–192,000,000)	210,000,000 (29,200,000–926,000,000)	155,000,000 (20,100,000–700,000,000)
NTM infection	192,000,000 (0–962,000,000)	1,030,000,000 (145,000,000–4,390,000,000)	307,000,000 (0–1,480,000,000)
Norovirus infection	27,600,000 (27,600,000–27,600,000)	26,800,000 (26,800,000–26,800,000)	4,530,000 (4,530,000–4,530,000)
Pseudomonas pneumonia	216,000,000 (14,400,000–958,000,000)	26,800,000 (931,000–142,000,000)	210,000,000 (17,500,000–935,000,000)
Pseudomonas septicemia	15,400,000 (394,000–86,000,000)	34,500,000 (459,000–196,000,000)	164,000,000 (7,520,000–806,000,000)
Salmonellosis, nontyphoidal	4,110,000 (75,500–22,800,000)	17,100,000 (635,000–83,800,000)	1,570,000 (16,000–9,980,000)
STEC infection, O157 serotype	1,840,000 (79,400–10,100,000)	710,000 (15,100–4,180,000)	129,000 (0–927,000)
STEC infection, non–O157 serotype	891,000 (836–5,600,000)	210,000 (0–1,620,000)	663,000 (541–4,270,000)
Shigellosis	2,660,000 (93,300–14,900,000)	118,000 (0–827,000)	695,000 (11,600–4,110,000)
Vibrio spp. Infection	3,990,000 (796,000–10,700,000)	35,400 (9–300,000)	79,300 (10–833,000)
Total Cost	1,070,000,000 (439,000,000–2,670,000,000)	1,390,000,000 (364,000,000–4,810,000,000)	871,000,000 (240,000,000–2,640,000,000)

*Estimates are rounded to 3 significant figures. Recreational water is used for recreational activities in treated (e.g., pools, hot tubs, and splash pads) or untreated (e.g., lakes, rivers, and oceans) venues; drinking water is used primarily for drinking but can also be used for hygienic activities, such as washing or showering, and can come from a public water system, private well, or commercially bottled sources; NRND water is used for purposes other than recreation or drinking (e.g., agriculture, industry, medical treatment, backcountry streams, or flood waters) (*1*). CrI, credible interval; NTM, nontuberculous mycobacteria; STEC, Shiga toxin-producing *Escherichia coli*.

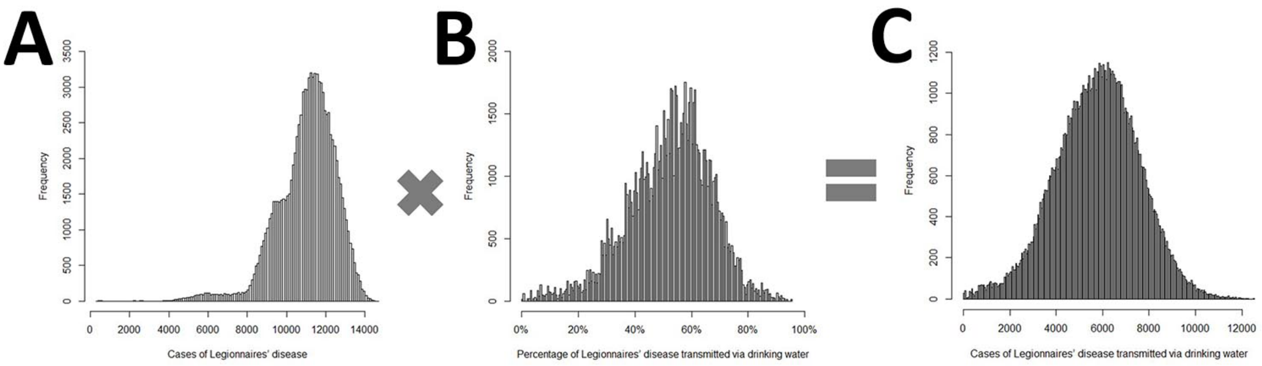

Appendix Figure. Example of probability modeling calculations performed for each pathogen, demonstrating probability distribution for the annual number of Legionnaires' disease cases transmitted through drinking water. A) Cases of Legionnaires' disease from all water exposure pathways. Dataset was 100,000 observations. Mean number of cases was 11,000 (rounded to 3 significant digits). The 2.5th percentile was 7,430, and the 97.5th percentile was 13,300. B) Percentage of Legionnaires' disease transmitted through drinking water. Dataset was 100,000 observations. Mean percentage of cases transmitted through drinking water was 52%. The 2.5th percentile was 19%, and the 97.5th percentile was 78%. C) Observations in each dataset from (A) and (B) were multiplied together to produce a 100,000-observation dataset that contains the estimated annual number of Legionnaires' disease cases transmitted through drinking water. The mean number of cases in the dataset was 5,760 (rounded to 3 significant digits). The 2.5th percentile was 2,030, and the 97.5th percentile was 9,160.
